# A Novel α4/7-Conotoxin QuIA Selectively Inhibits α3β2 and α6/α3β4 Nicotinic Acetylcholine Receptor Subtypes with High Efficacy

**DOI:** 10.3390/md20020146

**Published:** 2022-02-17

**Authors:** Liujun Wang, Xixi Wu, Xiaopeng Zhu, Dongting Zhangsun, Yong Wu, Sulan Luo

**Affiliations:** 1 Key Laboratory of Tropical Biological Resources of Ministry of Education, School of Pharmaceutical Sciences, Hainan University, Haikou 570228, China; wangliujun1995@163.com (L.W.); wuxixi@163.com (X.W.); zhangsundt@163.com (D.Z.); 2 Medical School, Guangxi University, Nanning 530004, China; biozxp@163.com

**Keywords:** α-conotoxin QuIA, nicotinic acetylcholine receptor, two-electrode voltage clamp, α3β2 nAChR, α6β4 nAChR

## Abstract

α6β4 nAChR is expressed in the peripheral and central nervous systems and is associated with pain, addiction, and movement disorders. Natural α-conotoxins (α-CTxs) can effectively block different nAChR subtypes with higher efficacy and selectivity. However, the research on α6β4 nAChR is relatively poor, partly because of the lack of available target-specific α-CTxs. In this study, we synthesized a novel α-4/7 conotoxin QuIA that was found from *Conus quercinus*. We investigated the efficacy of this peptide to different nAChR subtypes using a two-electrode voltage-clamp technique. Remarkably, we found α-QuIA inhibited the neuronal α3β2 and α6/α3β4 nAChR subtypes with significantly high affinity (IC_50_ was 55.7 nM and 90.68 nM, respectively), and did not block other nAChR subtypes even at a high concentration of 10 μM. In contrast, most α-CTxs have been determined so far to effectively block the α6/α3β4 nAChR subtype while also maintaining a similar higher efficacy against the closely related α6β2β3 and/or α3β4 subtypes, which are different from QuIA. In conclusion, α-QuIA is a novel α4/7-CTx, which has the potential to develop as an effective neuropharmacology tool to detect the function of α6β4 nAChR.

## 1. Introduction

Cone snails (*Conus*) are venomous marine predators that subdue prey and defend against invaders using their quick-acting venom [1]. The conotoxins (CTxs) are secreted in the long venom tubular ducts and venom glands of different cone snail species. The chemical nature of CTxs are small peptides that are generally 8–40 residues in length, and most of them contain two or three disulfides that target various ion channels, GPCRs, and transporters with high efficacy and selectivity [2]. CTxs can be divided into α, μ, μO, ω, ι, and δ subfamilies according to their different pharmacological targets [2,3,4]. Among them, α-CTxs specifically and potently inhibit different isoforms of neuronal nicotinic acetylcholine receptors(nAChRs), which improves our understanding of the physiological functions of these nAChRs [4,5]. Classic α-CTxs contain 8–20 residues, and their four cysteines are linked to form three isomers, including globular, ribbon and bead isomers. Most native α-CTxs exist as the globular isomer (CysI-CysIII/CysII-CysIV). α-CTxs can be further classified according to the number of residues in their intercysteine (loops). The most common loop subfamilies are the α3/5, α4/3, α4/4, and 4/7 subfamilies. Until now, α-4/7 CTxs have accounted for the vast majority of discovered α-CTxs, and they generally inhibit mammalian neuronal nAChRs [2,3,4,5,6].

Nicotinic acetylcholine receptors (nAChRs) are transmembrane pentameric proteins that belong to ligand-gated ion channels and are widely distributed in the central and peripheral nervous systems [7]. nAChRs are classified into muscle and neuronal subtypes arising from being expressed in muscle cells or neurons. For neuronal subtypes, homo- or heteropentamers of α (α1−α7, α9, α10) and/or β (β2−β4) subunits combine to produce a variety of neuronal nAChRs, such as α3β2, α3β4, α2β4, α4β2, α6β4, α6β2β3, α2β2, and α9α10 [8,9]. These subtypes are important in normal physiological functions and also involved in a range of disease states, including pain, addiction, epilepsy, autism, Alzheimer’s, and Parkinson’s diseases [9,10].

Noteworthy, the function of α6β4*nAChRs (asterisk indicates the possible presence of additional subunits) has not been adequately addressed. Previous research has reported this receptor regulating exocytosis in human adrenal chromaffin cells [11,12]. Besides, α6β4* nAChRs are highly expressed in dorsal root ganglia neurons, which are involved in pain [13,14,15]. Therefore, α6β4* nAChR has the potential to be developed as an attractive non-opioid therapeutic target for pain. However, the difficulty expressing α6β4* nAChRs in recombinant cells has delayed agonist or antagonist discovery. To increase the functional expression of α6 subunit, α6/α3 chimeric subunit was utilized to replace the α6 subunit, which means the N-terminal extracellular ligand-binding domain (LBD) of α6 subunit was chimeric with the transmembrane region of α3 subunit [16,17]. The α6/α3 receptor co-expressed with β4 subunit holds similar binding activity to natural α6β4 receptor and can be applied as a model for drug screening in vitro.

To date, there are only several α-CTxs that target α6β4* nAChRs, including VnIB, BuIA, and PIA. More importantly, most of them preferentially target rα6β2β3 over the closely related α6β4 nAChR except for VnIB [17,18,19,20]. In the present study, we described a novel α4/7-type conotoxin QuIA from *Conus quercinus*, which was previously identified using the high throughput transcriptomic sequencing method on an Illumina HiSeq4000 platform at BGI-Tech [21]. Here, we screened its electrophysiological activity and expected to find an α-CTx inhibiting nAChRs with high affinity and selectivity. Study results demonstrated α-CTx QuIA is a novel conopeptide that potently and preferentially antagonizes neuronal rα3β2 nAChR and rα6/α3β4 nAChRs, which can be developed as an effective pharmacological probe to detect the function of α6β4 nAChRs.

## 2. Results

### 2.1. Chemical Synthesis of α-4/7 QuIA

**Figure 1:**
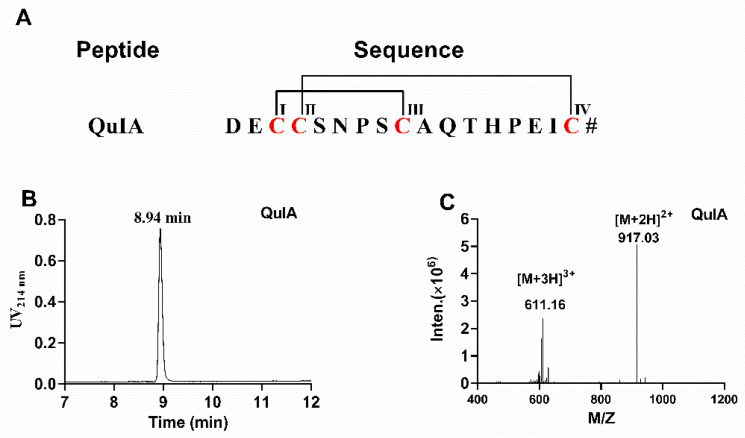
(**A**) The sequence of α-CTx QuIA and cysteines are marked in red. # indicates a C-terminal carboxamide modification. (**B**)The HPLC chromatogram of α-CTx QuIA, (**C**) ESI-MS analyses of folded α-CTx QuIA with an observed monoisotopic mass of 1832.10 Da.

The sequence and disulfide pattern of α-QuIA are shown in Figure 1A. Firstly, linear α-QuIA was successfully assembled using Fomc solid-phase peptide synthesis strategy. Functional groups in the side chains of the Cys were protected in pairs with orthogonal protecting groups. Specifically, CysI-CysIII was protected with acid-labile trityl (Trt) while CysII-CysIV was protected with acid-stable S-acetamidomethyl (Acm). The synthesized linear peptide was determined using analytical RP-HPLC and ESI-MS. After the linear peptide synthesis, the disulfide bonds were formed by a two-step oxidative folding approach. The first disulfide bond was formed using ferricyanide. Subsequently, the other disulfide bond between CysII-CysIV was produced by iodine oxidation. As shown in Figure 1, the purity of the oxidized QuIA is more than 95%, and ESI-MS was used to confirm the molecular weight of the folded QuIA. The synthetic molecular weight of QuIA (1832.1 Da) was consistent with theoretical molecular weight.

### 2.2. Effect of α-Conotoxin QuIA on Different nAChR Subtypes

**Figure 2:**
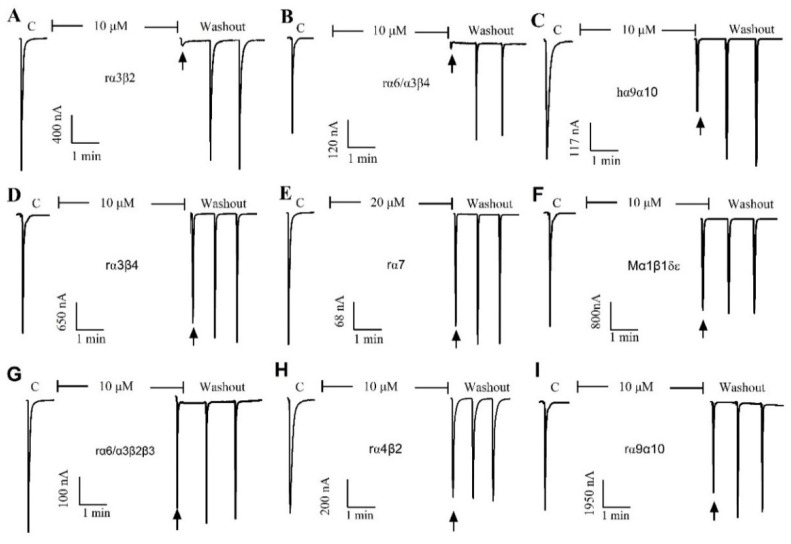
The QuIA blocks rα3β2, rα6/α3β4, and other nAChR subtypes differently at 10 μM concentration, and rα7 at 20 μM. The representative current-response is shown in a single oocyte, which was exposed to 10 µM α-CTx QuIA. The α-CTx QuIA almost completely blocked rα3β2 (**A**) and rα6β4 (**B**), but has no obvious inhibitory effect on other nAChR subtypes, such as hα9α10 (**C**), α3β4 (**D**), rα7 (**E**), Mα1β1δε (**F**), rα6/α3β2 (**G**), rα4β2 (**H**), and rα9α10 (**I**).

The pharmacological activity of QuIA was evaluated on 9 different human, rat, and mouse nAChRs subtypes expressed in *Xenopus laevis* oocytes utilizing a two-electrode voltage clamping technique. Typically, the peptide blocked less than 50% of 100 μM ACh-induced current at a concentration of 10 μM (rα7 for 20 μM), which indicated it had little or no inhibition on this subtype of nAChRs. The results displayed that QuIA has low or no inhibitory activity on a range of nAChRs subtypes, including hα9α10, mα1β1δε, rα7, rα6/α3β2β3, rα4β2, and rα9α10. In contrast, the ACh-evoked currents were blocked completely by QuIA on neural rα3β2 and rα6/α3β4 nAChRs (Figure 2).

**Figure 3:**
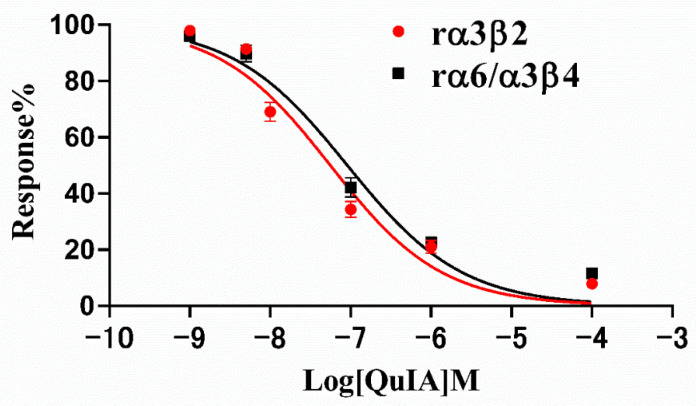
Response curves of α-CTx QuIA concentration to rα3β2 and rα6/α3β4 nAChRs. Exposure of rα3β2 and rα6/α3β4 nAChRs to QuIA. IC_50_ values are calculated as mean ± SD from 5–6 oocytes, respectively.

Therefore, we subsequently investigated the blocking effect of α-CTx QuIA on rα3β2 and rα6/α3β4 nAChRs, and their concentration-response curves are shown in Figure 3 The result displays dose-response curves of QuIA on rα3β2 and rα6/α3β4 nAChRs are similar (Figure 3). The IC_50_ values corresponding to each curve are 55.7 nM and 90.68 nM, respectively (Table 1). The results showed QuIA hold high efficacy for inhibiting rα3β2 and rα6/α3β4 subtypes.

### 2.3. CD Spectrum of QuIA

**Figure 4:**
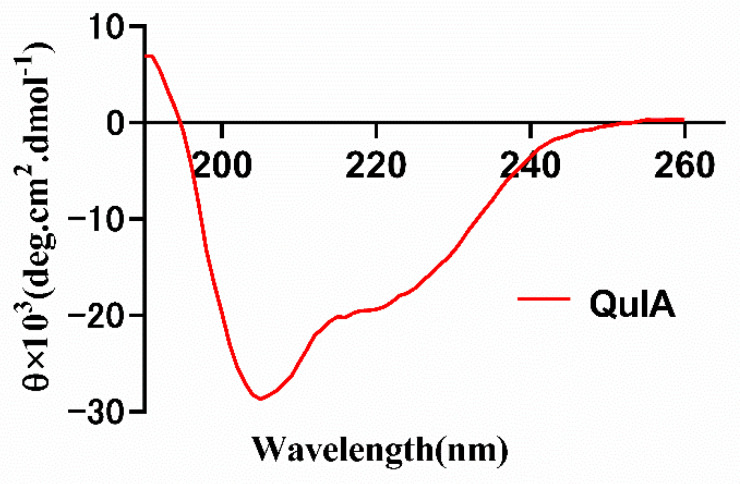
CD spectra of α-CTx QuIA in10 mM phosphate buffer solution.

Following the peptide synthesis, we investigated the conformation of QuIA using circular dichroism (Figure 4). The results show there are two strong negative peak wavelengths at around 208 and 220 nm, and a positive peak at around 190 nm, which indicates QuIA contains an α-helix motif. Then, the secondary structure of QuIA was calculated by the CDSSTR program, the α-helix and β-sheet motifs content were 21.0% percentage. These results display the conformation of QuIA is similar to other α-4/7-CTxs.

## 3. Discussion

Owing to high target affinity and selectivity, relatively small size, and structural stability, CTxs are thought as ideal probes to detect the function of ion channels and peptide drug candidates [3]. The clinical application of CTxs in analgesia has been a remarkable success. ω-MVIIA, an N-type calcium channel blocker, which was purified from Conus magus, has been approved by the FDA for the treatment of severe chronic pain. Other CTxs, such as χ-MrIA, ω-CVID, contulakin G, and α-Vc1.11 have also entered clinical trials. More recently, α-CTxs antagonizing α6β4, α3β2, α7, and α9-containing nAChRs have been identified as promising new leads to treat neuropathic pain [15].

To date, along with advances in sequencing technologies, the venom duct transcriptomes of nearly 30 species of *Conus* have been sequenced [3,22,23]. As a result, a wide variety of conotoxins were found using this strategy, but the targets of most of them have not been tested yet. Therefore, identifying the target and activity of these unknown conotoxins will be very valuable and interesting work. Here, α-CTx QuIA was found using RNA-seq sequencing from *Conus quercinus,* which is identical with a CTx-Eb1.2 cloned from *Conus ebraeus* (GenBank: AGK23171.1). However, the functional property of QuIA was still unknown. The QuIA belongs to the α-4/7 subfamily and contains two disulfide bonds. More importantly, it contains a conserved Ser-X-Pro-X motif (where X denotes any different amino acid) in the loop I domain, which is important for α-4/7 α-CTx binding activity to neuronal nAChR subtypes. According to these characteristics, we speculated QuIA could also block some kind of neuronal nAChR subtypes, and the results of electrophysiological experiments confirmed our hypothesis.

α3β2 nAChRs were found in the dorsal root ganglia, which is associated with regulating pain awareness [24]. α6β4* is another type of nAChR also highly expressed by the dorsal root ganglia, which is an attractive target for treating neuropathic pain [13,15,25]. Furthermore, α6β4* nAChR is also found in the hippocampus regulating the secretion of norepinephrine, which is closely related to learning and memory [15,26]. Here, α-CTx QuIA is an antagonist with a high affinity for α6β4 and α3β2 subtypes. Therefore, it can be developed as a pharmacological probe detecting the functions of α3β2 and α6β4 nAChR subtypes. In addition, α-CTx QuIA has the potential to be a drug candidate for the unmet medical needs of chronic neuropathic pain.

α-CTxs is a rich source of antagonists targeting numerous nAChRs. α-4/7 CTxs generally block the different neuronal nAChRs [4,5,22]. In this report, α-QuIA has high activity on both α3β2 and α6β4 nAChRs, and it does not block other nAChR subtypes. Previous studies have found several α-CTxs target α3β2 subtypes with high affinities, such as LtIA, MII, OmIA, GIC, and LvIA, and some of them also antagonize other neuronal nAChRs [27,28,29,30]. However, only a few of the α-CTxs targeting α6*nAChRs are reported (Table 2), and most of them inhibit α6β2β3 and α3β2 nAChRs. In contrast, α-CTx VnIB was the only α-CTx that selectively and potently blocks the α6β4 nAChR [18]. Similarly to QuIA, α-CTx PeIA also blocks α3β2 and α6/α3β4 nAChRs, and a recent study demonstrated α-CTx PeIA was redesigned using the computational method and one of its analogue (PeIA-5667) displayed a more than 250-fold increase in efficacy for inhibition of rat α6/α3β4 nAChRs [31].

In this study, we identified a novel α4/7-CTx QuIA as an antagonist of rα3β2 and α6/α3β4 nAChRs with high efficacy. This peptide can be developed as a molecular probe to deeply detect the pathological molecular mechanism of pain related to α3β2 and α6/α3β4. More importantly, the QuIA may inform the design of therapeutic ligands that target α6β4 nAChRs for the treatment of neuropathic pain. Next, we will modify α-CTx QuIA using amino acids substitution strategy, and the goal of the modification is to acquire some analogues specifically targeting human or rat α6β4 nAChRs, which is hopeful to be developed as a promising new drug candidate for the treatment of neuropathic pain.

## 4. Materials and Methods

### 4.1. Materials

Rink amide-MBHA resin, triisopropylsilane (TIPS), diisopropylethylamine (DMF), Fmoc (L) amino acid derivatives N, N′-Diisopropylcarbodiimide (DIC), and all synthesis reagents were purchased from GL Biochem Ltd. (Shanghai, China). HPLC grade Trifluoroacetic acid (TFA), acetonitrile (ACN), and MeOH are from Sigma–Aldrich; Oxyma was purchased from CEM corporation. Clones of rat (r) α2, α3, α4, α7, and β2, β3, β4, as well as the mouse (m) α1, β1, δ, ε cDNAs were generously provided by S. Heinemann (Salk Institute, La Jolla, CA, USA). Clones of rα9 and rα10 were kindly provided by A.B. Elgoyen (Instituto de Investigaciones en Ingeniería Genética y Biología Molecular, Buenos Aires, Argentina). Rα6/3 chimera clone was generously provided by J. E. Garrett (Cognetix, Inc., Salt Lake City, UT, USA). *Xenopus Laevis* was obtained from Nasco (Fort Atkinson, WI, USA). Waters 2695 preparative high-performance liquid chromatography (RP-HPLC), analytical high-performance liquid chromatography (Waters Corp., Milford, MA, USA); Dual-electrode voltage Clamp system (Axon 900A, Molecular Device, Sunnyvale, CA, USA); Digidata 1550B digital-to-analog converter, Axon 900A amplifier and Cell Works software (Molecular Devices Corp., Sunnyvale, CA, USA); Alpha 1–4L freeze dryer (Marin Christ Company, Osterode, Germany), Jasco J-810 circular dichroic spectrometer (JASCO, Hachioji, Japan).

### 4.2. Peptide Synthesis

The linear peptide was synthesized using the Fmoc strategy on the CEM Liberty Blue peptide synthesizer. The crude peptide was separated and purified by preparative reverse liquid chromatography. A two-step oxidation procedure was carried out as the previous study [28]. In brief, oxidative buffer (20 mM potassium ferricyanide (K_3_(Fe(CN)_6_)), 0.1 mM Tris, pH 7.5) was used to form the first pair of a disulfide bond. The purified linear peptide was slowly added to the reaction solution of potassium ferricyanide at room temperature for 45 min to form the monocyclic peptide between Cys-I and Cys-III. After that, the oxidized products in one step were slowly added into iodine reaction solution (10 mM, I_2_ solution, Water-TFA-ACN, 20:1:8, *v*/*v*) for 10 min under the atmosphere of nitrogen. Then the Acm-group was removed, and the second disulfide bond was formed between Cys-II and Cys-IV. After the completion of oxidation, vitamin C was added to terminate the I_2_ oxidation reaction. Finally, the molecular weight of folded QuIA was determined by ESI-MS, and the purity and quantity of the peptide were assessed by analytical RP-HPLC.

### 4.3. Establishment of nAChRs Model and cRNA Preparation and Injection

The plasmids containing different kinds of nAChR subunits were transferred into DH5α competent cells, then they were extracted and digested with the appropriate restriction enzymes. The linearized DNA templates were purified, and their concentration and purity were determined by the gel electrophoresis method. The Invitrogen™ Ambion™ mMESSAGE mMACHINE™ T7 Transcription Kit was used for transcription, and the transcribed RNA was purified to obtain the final cRNA. RNA concentration and purity were assessed using the gel electrophoresis method. The pre-prepared RNAs of different subunits were mixed and injected into *Xenopus laevis* oocytes as described previously [40]. After injection, oocytes were cultured in ND_96_ solution (96 mmol/L NaCl, 2 mmol/L KCl, 1.8 mmol/L CaCl_2_, 1 mmol/L MgCl_2_, 5 mmol/L HEPES, pH 7.1–7.7) containing 1% penicillin (10 μg/mL), 1% streptomycin (10 μg/mL), 1% gentamicin (100 μg/mL) at 17 °C for 2–5 days, then the currents were detected by the TEVC. In this experiment, the animal assay was in accordance with the Guide for the Care and Use of Laboratory Animals and approved by the Ethics Committee of Hainan University.

### 4.4. Electrophysiology

The oocytes injected with cRNA were placed in a 50 μL cell tank and fixed. The flow rate of ND96 added with BSA was 2 mL/min and the concentration of agonist acetylcholine (ACh) was 100 μM. The current response to ACh was tested using the two electrode voltage clamp amplifier Axon 900A (Molecular Device, Sunnyvale, CA, USA), and the holding potential was −70 mV. The opening of channels were activated by ACh and the inward current was produced. ND_96_ was used as blank control. The ACh-induced control current was repeated three times to obtain the average peak current. Each concentration of QuIA was tested on five to six oocytes. A scan corresponding to the Mα1β1δε and rα9α10 subtypes had 1-s pulses of 10 µM ACh, 200 µM ACh for the rα7 subtype, and 100 µM ACh for all other subtypes. Peak current amplitudes were measured and analyzed by Clampfit 10.2 software (Molecular Devices, Sunnyvale, CA, USA) before and after peptide incubation. All records were made at room temperature (20–25 °C). The detailed analysis of this assay was carried out mainly as described previously [40].

### 4.5. Data Analysis

The response of the α-CTx QuIA was defined as the peak current amplitude at the ACh induced steady-state current, and the “% response” was calculated by dividing this value by the toxin pre-baseline. Each data point in the dose-response curve represents the mean ± SEM of at least three oocytes. The dose-response data were fit to the nonlinear regression equation: response (%) = 100/{1 + ([Toxin]/IC_50_)^nH^}, where nH is the Hill coefficient and IC_50_ value is calculated for the concentration of antagonist producing a half-maximal inhibition using GraphPad Prism 6.0(GraphPad Software, San Diego, CA, USA).

### 4.6. Determination of the Secondary Structure Using Circular Dichroism

Circular dichroism (CD) was adopted to verify the secondary structure of QuIA. CD spectra were obtained using a Jasco J-810 circular dichroism spectrometer. The experiment was carried out at room temperature under a nitrogen atmosphere. The scanning speed was 50 nm/min with a resolution of 0.5 nM. The absorbance was measured in the far ultraviolet region (190–260 nm) with a 0.1 cm path length cell. The results were recorded by four scans. The peptide QuIA was dissolved in 20 mM ammonium bicarbonate buffer at pH = 7. CD data in ellipticity was converted to mean residue ellipticity ((θ)R) using the equation: (θ)R = θ/(10 × C × Np × l) where θ is the ellipticity in millidegrees, C is the peptide molar concentration (M), l is the cell path length (cm), and Np is the number of peptide residues.

## Figures and Tables

**Table 1:** The IC_50_ and Hill slope values of α-CTx QuIA inhibiting different nAChR subtypes.

Subtype	IC_50_(nM) ^a^	Hill Slope ^a^
rα3β2	55.7 (39.71–79.78)	0.8 (0.7–1.2)
rα6/α3β4	90.68 (67.72–122.0)	0.8 (0.6–1.1)
rα3β4	>10,000 ^b^	ND
rα7	>20,000 ^b^	ND
mα1β1δɛ	>10,000 ^b^	ND
rα4β2	>10,000 ^b^	ND
rα9α10	>10,000 ^b^	ND
hα9α10	>10,000 ^b^	ND
rα6/α3β2β3	>10,000 ^b^	ND

^a^ Values in parentheses are a 95% confidence interval (C.I.). ^b^ Less than 50% blocking at 10 µM. All receptors are of rat (r) or human (h) origin, except α1β1δε, which is of mouse (m) origin. ND, not determined.

**Table 2:** α-CTx QuIA and other α-4/7 CTxs the target α6* nAChR according to previously published data.

α-CTx	Organism	Sequence	Target ^a^	Ref.
QuIA	*C.quercinus/* *C. ebraeus*	DE**CC**SNPS**C**AQTHPEI**C**#	rα3β2 ≈ rα6β4	This work
MII	*C.magus*	G**CC**SNPV**C**HLEHSNL**C**#	rα6β2β3 > rα3β2 > rα6β4	[32,33]
TxID	*C.textile*	G**CC**SHPV**C**SAMSPI**C**#	rα3β4 > rα6β4 ≫ rα2β4	[34]
VnIB	*C. ventricosus*	GG**CC**SHPV**C**YTKNPN**C**G#	rα6β4 > rα3β4 ≫ rα6β2β3	[18]
PIA	*C. purpurascens*	RDP**CC**SNPV**C**TVHNPQI**C**#	rα6β2β3 > rα6β4 ≈ rα3β2 > rα3β4	[17]
BuIA	*C. bullatus*	G**CC**STPP**C**AVLY**C**#	rα6β2β3 > rα6β4 > rα3β2 > rα3β4	[19,20]
RegIIA	*C. regius*	G**CC**SHPA**C**NVNNPHI**C**#	rα3β2 > rα3β4 ≈ rα6β2	[35,36]
PeIA	*C. pergrandis*	G**CC**SHPA**C**SVNHPEL**C**#	rα3β2 ≈ rα6/α3β4 ≈rα6/α3β2β3 > rα6β4	[37,38,39]

^a^ All the targets are rat nAChRs unless otherwise indicated. # C-terminal carboxamide.

## Data Availability

The data that support the findings of this study are available from the corresponding author upon reasonable request.
